# Electrical Vestibular Stimuli to Enhance Vestibulo-Motor Output and Improve Subject Comfort

**DOI:** 10.1371/journal.pone.0084385

**Published:** 2014-01-02

**Authors:** Patrick A. Forbes, Christopher J. Dakin, Anoek M. Geers, Martijn P. Vlaar, Riender Happee, Gunter P. Siegmund, Alfred C. Schouten, Jean-Sébastien Blouin

**Affiliations:** 1 Department of Biomechanical Engineering, Faculty of Mechanical, Maritime and Materials Engineering, Delft University of Technology, Delft, The Netherlands; 2 Laboratory of Biomechanical Engineering, Institute for Biomedical Technology and Technical Medicine (MIRA), University of Twente, Enschede, The Netherlands; 3 School of Kinesiology, University of British Columbia, Vancouver, British Columbia, Canada; 4 Brain Research Center, University of British Columbia, Vancouver, British Columbia, Canada; 5 Institute for Computing, Information and Cognitive Systems, University of British Columbia, Vancouver, British Columbia, Canada; 6 MEA Forensic Engineers & Scientists, Richmond, British Columbia, Canada; McGill University, Canada

## Abstract

Electrical vestibular stimulation is often used to assess vestibulo-motor and postural responses in both clinical and research settings. Stochastic vestibular stimulation (SVS) is a recently established technique with many advantages over its square-wave counterpart; however, the evoked muscle responses remain relatively small. Although the vestibular-evoked responses can be enhanced by increasing the stimulus amplitude, subjects often perceive these higher intensity electrical stimuli as noxious or painful. Here, we developed multisine vestibular stimulation (MVS) signals that include precise frequency contributions to increase signal-to-noise ratios (SNR) of stimulus-evoked muscle and motor responses. Subjects were exposed to three different MVS stimuli to establish that: 1) MVS signals evoke equivalent vestibulo-motor responses compared to SVS while improving subject comfort and reducing experimentation time, 2) stimulus-evoked vestibulo-motor responses are reliably estimated as a linear system and 3) specific components of the cumulant density time domain vestibulo-motor responses can be targeted by controlling the frequency content of the input stimulus. Our results revealed that in comparison to SVS, MVS signals increased the SNR 3–6 times, reduced the minimum experimentation time by 85% and improved subjective measures of comfort by 20–80%. Vestibulo-motor responses measured using both EMG and force were not substantially affected by nonlinear distortions. In addition, by limiting the contribution of high frequencies within the MVS input stimulus, the magnitude of the medium latency time domain motor output response was increased by 58%. These results demonstrate that MVS stimuli can be designed to target and enhance vestibulo-motor output responses while simultaneously improving subject comfort, which should prove beneficial for both research and clinical applications.

## Introduction

Electrical vestibular stimulation is a non-invasive technique for investigating vestibular function during balance or locomotor tasks [1]–[4]. Electrical current delivered through electrodes placed bilaterally over the mastoid bones modulates the firing rate of the vestibular nerve [5] and induces an artificial sensation of motion. The perceived motion is accompanied by both muscular responses and whole-body postural adjustments [1], [2], [6]. The majority of studies using electrical vestibular stimulation apply square-wave transient stimuli [2], [7]–[9]. Recently, the use of stochastic vestibular stimulation (SVS) signals has received attention due to the advantages it possesses over square-wave stimuli. Primarily, SVS provides more detailed insight into vestibular contributions to postural control by examining the frequency behaviour between SVS and muscular or full body postural responses [3], [10], [11]. The cross-correlation between SVS and muscle activity is equivalent to the time response of square-wave stimuli [12], and the resultant motor output (i.e. force) can be used to estimate response direction [13]. In addition, SVS signals offer several experimental advantages over its square wave equivalent: minimizing anticipatory effects [14], shortening experimental durations [12] and improving signal-to-noise ratios [15].

A significant limitation of both square-wave and stochastic vestibular stimulation methods is the relatively small muscle and balance responses induced by these stimuli [7], [8]. During SVS for example, coherence between the input stimulus and output lower limb muscle activity is low, and only 5–10% of the oscillatory muscle behaviour can be attributed to the input stimulus [3], [11], [12]. Although response magnitudes and coherences can be improved by increasing the amplitude of the applied stimulus, high current amplitudes decrease subject comfort and are often perceived as noxious or painful [8]. This is particularly important when using electrical vestibular stimulation as a clinical tool to evaluate neurologically deficient patients [16]–[21]. In clinical settings it is essential to limit the level of discomfort to avoid confounding effects of poor task performance and fatigue, and to obtain a reliable measurement for diagnostic and treatment purposes.

Multisine signals, which consist of multiple sinusoids, provide a potential solution to these problems because they can be optimized to maximize signal-to-noise ratios, thereby minimizing required input amplitudes while maintaining reliable estimates of reflex behaviour [22], [23]. Furthermore, multisine signals can be designed to concentrate power at a precise number of frequencies within the bandwidth of interest, which can be beneficial to target specific components of the vestibulo-motor responses [11], [24], [25]. Therefore, the aim of the current study was to develop multisine vestibular stimulation (MVS) signals that ensure reliable estimates of reflex responses while simultaneously minimizing the subject's perception of discomfort caused by the stimulation. The results show that MVS signals can achieve increased signal-to-noise ratios and improve subject comfort compared to SVS. Consequently, MVS offers the potential advantage of reducing the applied current amplitude and experimentation time required to estimate vestibulo-motor responses.

## Methods

### Subjects

Eight healthy subjects (7 males, 1 female; age 24–48 yrs; mass 74±10 kg; height 1.74±0.12m; mean ± SD) with no self-reported history of neurological disorders or injuries participated in these experiments. The protocol was explained prior to the experiment and all subjects gave written informed consent. The experiment conformed to the Declaration of Helsinki and was approved by the University of British Columbia's Clinical Research Ethics Board.

### Vestibular stimuli

Vestibular stimulation was applied to the subjects using carbon rubber electrodes (∼9 cm^2^) in a binaural bipolar electrode arrangement. The electrodes were coated with Spectra 360 electrode gel (Parker Laboratories, Fairfield, NJ, USA) and secured over the subject's mastoid processes with an elastic headband. The stimuli were delivered as an analogue signal via a digital-to-analogue board (PXI-6289, National Instruments, Austin, USA) to an isolated constant-current stimulator (STMISOL, Biopac, Goleta, CA, USA). The signals were generated offline using Matlab (Mathworks Inc., Natick, MA, USA) and the same signals were presented to all subjects.

**Figure 1:**
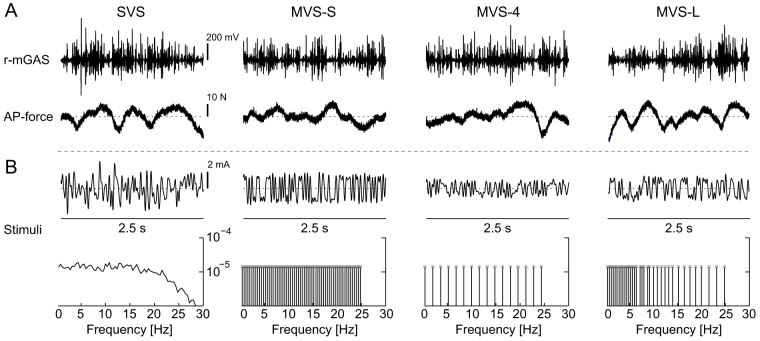
Raw EMG data and applied stimuli during each condition (SVS, MVS-S, MVS-4 and MVS-L). A: 2.5 s of raw data depicting muscle EMG (r-mGAS) and anterior-posterior directed force (+ve anterior). B: 2.5 s of applied stimuli and power spectra for each of the stimulus conditions. Circles in MVS plots represent exact frequencies chosen to be included in each signal. SVS, stochastic vestibular stimulation; MVS, multisine vestibular stimulation; r-mGAS, right medial gastrocnemius; AP, anterior-posterior.

Subjects were exposed to four 90 s signals of varying spectral designs with a bandwidth of 0–25 Hz, which is sufficient to capture the bandwidth of vestibular reflexes in lower limb muscles [12]. The first signal was a stochastic (SVS) filtered white noise (fourth-order Butterworth lowpass at 25 Hz), used here as a control condition and for comparison to previous studies [11], [12]. The other three signals consisted of different multisine signals. All four signals were designed with similar power amplitudes (Fig. 1) at each excited frequency to provide a standardized comparison of multisine vestibular stimulation (MVS) signals to the control SVS signal.

Multisine signals are advantageous over white noise stimuli (i.e. SVS) because they are deterministic and thereby avoid spectral leakage [26]. They can be designed to include a limited and precise number of frequency points, thus increasing the relative power per frequency. In addition, the total signal power can be increased without changing the signal peak amplitude by minimizing the crest factor [22], [23], [26], defined as:
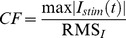
(1)in which RMS*_I_* denotes the RMS of the applied current *I_stim_(t)*.

All MVS signals were crested and designed to have a period length of 2.5 seconds, providing a frequency resolution of 0.4 Hz, and repeated 36 times in each 90 s trial. The first MVS signal (MVS-S) was designed to excite all 62 frequencies from 0.4–24.8 Hz. The MVS-S was the closest multisine equivalent to the SVS (hence the −S in MVS-S) and was expected to increase signal-to-noise ratios because of the cresting process and to improve subject comfort because of the reduced signal amplitude. The remaining two MVS signals were designed to excite a limited set of frequencies from 0.4–24.8 Hz including: 1) a uniform distribution of every fourth frequency (MVS-4; 16 frequencies) and 2) a logarithmic distribution focusing power at lower frequencies (MVS-L; 35 frequencies). The MVS-4 was used to evaluate potential harmonic distortions generated by system nonlinearities during multisine stimulation (see *Data collection and signal analysis*). MVS-L was used to demonstrate the versatility of MVS signals to target specific components of the vestibulo-motor responses while characterizing the entire bandwidth of the frequency response (i.e. 0.4–24.8 Hz). Specifically, by concentrating power at low frequencies, MVS-L was expected to enhance the medium latency components of both the muscle and anterior-posterior (AP) force responses [11], [27], which are thought to be primarily influenced by a net canal signal [24]. The signals were designed with equal absolute power per frequency, therefore all four signals had different peak and root-mean-square (RMS) amplitudes: 5.00/1.12 mA (SVS), 1.95/1.17 mA (MVS-S), 1.16/0.60 mA (MVS-4) and 1.71/0.89 mA (MVS-L) (peak/RMS amplitude; see Fig. 1).

### Protocol

Subjects stood on a force plate (Bertec 4060–80; Bertec, Columbus, OH, USA) with their feet 2–3 cm apart (as measured at the medial malleoli). Subjects were instructed to stand relaxed, lean forward slightly, close their eyes, hold their arms by their sides and rotate their torso and head axially to the left (i.e. leftward yaw). Lasers attached to their torso (T2 level) and head were used to maintain torso yaw at 30° and head yaw at 90° relative to the feet (60° relative to the torso). In addition, the head was rotated in extension such that the Reid's plane was angled 18° up from the horizontal. In this position the postural response to electrical vestibular stimulation is maximized in the AP direction [6], [28] along the line of action of the right plantar flexor muscles. Each 90 s stimulus (SVS, MVS-S, MVS-4 and MVS-L) was applied twice in a random order. The MVS and SVS signals used in this study, along with the software used to generate them, have been made available for download at http://www.3me.tudelft.nl/nmc and included here as File S1.

### Data collection and signal analysis

Surface electromyography (EMG) was collected from the right medial gastrocnemius (r-mGAS) muscle. EMG signals were amplified (2000×; NeuroLog, Digitimer, Hertforshire, UK) and band pass filtered before digitization (10–1000 Hz). EMG, force plate and vestibular stimuli signals were digitized and recorded at 2 kHz via a digital acquisition board (PXI-4495; National Instruments, Austin, TX, USA) using a custom LabVIEW software program (National Instruments, Austin, TX, USA). Horizontal AP forces measured from the force plate were used to describe the balance responses of the subjects (Fig. 1) [7], [11], [13].

Repeated trials within each subject were concatenated to create 180 s data records. Data from all subjects (EMG and force) were then concatenated to create a single pooled data set for each condition. Both the individual and pooled EMG data were full-wave rectified and the autospectra for the input stimuli, muscle activity and AP force as well as the cross-spectra of the input stimuli with the muscle activity and AP force were calculated. Data were sectioned into 2.5 s segments prior to calculating the auto and cross-spectra, and then the resulting spectra were averaged in the frequency domain. This yielded a frequency resolution of 0.4 Hz for all spectra.

Coherence functions were estimated to indicate how much of the output power (EMG and AP-force) was related to the input power. The 95% confidence limits for coherence were derived from the number of disjoint 2.5 s segments (individual subject data: 72; pooled data: 576) to determine the frequencies where coherence was significantly different from 0 [29]. Coherence ranges from 0 to 1, where for the SVS signal 1 indicates a linear, noise free, input-output relationship [26]. For periodic MVS signals however, harmonic noise generated by nonlinearities in the system can bias the coherence estimates at the stimulated frequencies [30] and distort linear estimates of the system dynamics (i.e. gain, phase and cumulant densities, see below). As a result, no formal comparisons of the coherence were made between the SVS and MVS signals.

Nevertheless, to ensure that MVS signals did not distort the linear estimates of system dynamics, the magnitude of nonlinear distortions were estimated using the techniques of Pintelon and Schoukens [26] during the MVS-4 stimulus condition. Nonlinear distortions were evaluated at non-stimulated even and odd harmonics of the stimulated frequencies using the fast Fourier transform (FFT) of the pooled rectified-EMG and AP force. For this analysis, data were sectioned into 5 s segments to provide double the frequency resolution, thus allowing for the comparison of power at non-stimulated harmonic frequencies (i.e. 0.8, 1.2, 1.6, … Hz), which include noise and nonlinear distortions, and non-stimulated non-harmonic frequencies (i.e. 0.6, 1.0, 1.4, 1.8,… Hz), which include noise only. Assuming the system is linear, power at non-stimulated harmonic frequencies (i.e. noise and nonlinear distortions) should be similar to power at non-stimulated non-harmonic frequencies (i.e. noise). To test for a significant influence of nonlinear distortions, we compared the mean FFT squared (power) to the variance of the FFT at non-stimulated frequencies. The output signal (rectified-EMG and AP force) is influenced by nonlinearities if the power at non-stimulated frequencies significantly exceeds the variance. The test was performed at each frequency by comparing the ratio of the FFT squared to its variance to the 95^th^ percentile of an F-distribution with 2×2N-2 degrees of freedom (N is the number of segments of the pooled data: 576) [26], [31]. By demonstrating little to no influence of system nonlinearities at non-stimulated harmonic frequencies during the MVS-4 stimulus (see Results), it was possible to confirm that nonlinearities did not substantially affect the system dynamics estimated at stimulated frequencies.

Gain and phase estimates for stimulus-EMG and stimulus-AP force were then computed to identify the dynamic behavior of vestibulo-motor responses. Gain- and phase-frequency response functions were estimated by dividing the cross-spectral density of stimulus-EMG and stimulus-AP force by the autospectral density of the stimulus. We expected that frequency responses would be similar across stimuli since phase responses during SVS are equivalent to responses obtained during stimulation by single sinusoids [27].

Cumulant density functions were estimated using the individual and pooled data to evaluate the time domain correlation between input stimuli and output muscle activity and AP force. Cumulant densities were calculated by taking the inverse Fourier transform of the cross-spectrum [29] and were normalized by the product of the vector norms of the input and output to provide coefficients of correlation (r) ranging between −1 and +1. Normalization allowed comparison between the individual subject responses [11]. For statistical analysis, dependent variables were extracted from cumulant density functions using individual subject data, whereas the cumulant density functions estimated using the pooled data were used for illustrative purposes only. Although the correlated responses have no physical units, they possess similar spatial and temporal characteristics to EMG [11], [12] and force [13] in response to trigger-averaged transient stimulation. Both EMG and AP force cumulant density responses exhibit a biphasic pattern with two opposing peaks, defined as short and medium latency components. By convention, anode right/cathode left (ARCL) currents represent a positive vestibular signal. Thus, for stimulus-EMG relationships, positive cumulant density values indicate excitation of muscle activity due to ARCL currents, and for stimulus-force relationships, positive values indicate force applied in an anterior direction on the feet in response to ARCL currents. A normalized 95% confidence interval was calculated for each subjects cumulant density function to indicate where responses were significant [29]. The magnitude and timing of the cumulant density peaks were used to evaluate differences between SVS and MVS stimuli. Specifically, we expected that (1) MVS-S would elicit response latencies and magnitudes that would not differ compared to SVS but with a decreased intensity and unpleasant perception of the stimulus, and (2) MVS-L would elicit reduced short latency and increased medium-latency response magnitudes compared to SVS due to the concentration of power at low frequencies, also with a decreased intensity and unpleasant perception of the stimulus. We limited our statistical analysis to comparisons (SVS vs. MVS-S and SVS vs. MVS-L) of peak magnitude and timing to assess these predictions using paired t-tests.

To validate the technique further, two additional analyses were performed. First we examined the minimum number of disjoint segments required for the estimated short and medium latency peaks in both the stimulus-EMG and stimulus-AP force cumulant density functions to exceed their confidence intervals. Because the magnitude of the cumulant density responses depends on the frequency content of the input stimuli [11], [27], the analysis was limited to SVS and MVS-S (and compared using paired t-tests) since the frequencies contributing to MVS-4 and MVS-L strongly deviate from SVS. Second, we estimated the total signal-to-noise ratio (SNR) for each subject per stimulus condition for both the output EMG and AP force. The SNR was calculated per frequency as the ratio of the magnitude of the mean FFT to the standard deviation of the FFT across all segments (n = 72). Total SNR was defined as the mean SNR using only frequencies excited by each stimulus. Comparisons between all stimuli were made using repeated-measures ANOVA, and pairwise comparisons of MVS conditions with SVS were made using a Bonferroni correction for multiple comparisons. A significance level of α = 0.05 was used for all analyses.

Finally, to compare the subjective perceptions of the applied stimuli, subjects were asked to rate the stimulus after each trial according to three psychophysical criteria: intensity, unpleasantness and imbalance. Each criterion used a visual analogue scale (VAS) comprised of a 10 cm printed horizontal line without tick marks, where the left side represented not intense at all, not unpleasant at all and no imbalance, and the right side represented most intense feeling ever, most unpleasant feeling ever and complete imbalance, respectively. Because the stimuli were designed to have equivalent power per frequency and therefore varied in amplitude, we expected that signals with the lowest peak amplitude would elicit the lowest intensity, unpleasantness and imbalance scores. VAS scores were compared across signals using three separate repeated-measures ANOVAs, and pairwise comparisons of MVS stimuli with SVS were made using paired t-tests with a Bonferroni correction for multiple comparisons. We chose not to evaluate pain because continuous vestibular stimulation (SVS and MVS) is more often associated with a ‘tapping’ and ‘irritating’ cutaneous feeling or a slightly ‘nauseating’ perception [12], [32], as opposed to the explicitly ‘sharp, local cutaneous pain’ associated with transient square wave stimuli [8].

## Results

### Linearity assessment of electrically evoked vestibulo-motor responses

**Figure 2:**
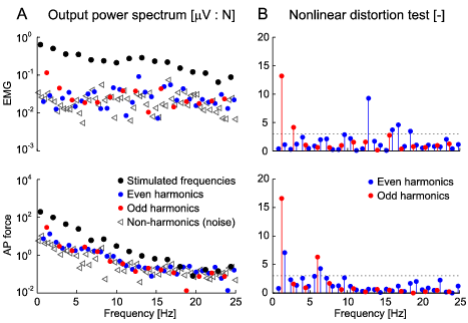
Assessment of system linearity using EMG and AP force data. A: pooled (n = 8) power spectrum of EMG (top plots) and AP force (bottom plots) during the MVS-4 stimulus condition. Power at even and odd harmonic frequencies are similar to power at non-harmonic frequencies. B: nonlinear distortion test during the MVS-4 stimulus condition. Significant nonlinear distortions are detected when points exceed the horizontal segmented line representing the significance level of the F-distribution (α = 0.05).

For the FFT estimated from the MVS-4 condition (Fig. 2A), the power at non-stimulated even and odd harmonics of the stimulated frequencies (blue and red dots, Fig. 2A) was similar in magnitude compared to the noise at non-stimulated non-harmonic frequencies (open triangles, Fig. 2A). These results indicated that nonlinearities within the system did not elicit harmonic distortions that were apparent relative to the level of noise within the system. This was confirmed by the ratio of the FFT squared to its variance remaining below the significance level at the majority of harmonic frequencies (>87%) (Fig. 2B). At frequencies where significant nonlinear distortions were detected (6 harmonics in EMG and 4 harmonics in AP force), the power was no more than 25% of the power at adjacent stimulated frequencies. At higher frequencies, the power at stimulated frequencies was similar to the power at non-stimulated harmonic and non-harmonic frequencies, indicating that the stimulated frequencies were not passing through, likely as a result of the low-pass filtering that occurs in both EMG and force responses [10], [11]. Overall, these results support the use of linear analysis methods and allow us to compare gain, phase and cumulant density responses between SVS and all MVS conditions.

### Vestibulo-motor response characterization: SVS vs. MVS

MVS signals improved the SNR and reduced the number of segments needed to estimate the cumulant density. The total SNR for EMG was 3.6, 4.1 and 4.5 times larger for the MVS-S, MVS-4 and MVS-L stimuli, respectively, when compared to the SVS stimulus (F_3,21_ = 82.9, P<0.001) (see Table 1). Similarly, the total SNR for AP force was 4.6, 5.4 and 6.3 times larger for the MVS-S, MVS-4 and MVS-L stimuli, respectively, when compared to the SVS stimulus (F_3,21_ = 82.9, P<0.001) (see Table 1). The minimum number of segments needed for the cumulant density peaks to exceed the 95% confidence interval was reduced using MVS-S in comparison to SVS. The stimulus-EMG cumulant density for the MVS-S signal required 15% of the segments used for the SVS signal (t_7_ = 3.7, P = 0.007; 1.5±0.5 vs. 10.5±6.9) and 36% for the stimulus-AP force cumulant density (t_7_ = 2.4, P = 0.049; 7.1±0.5 vs. 19.4±12.1). In fact, for all individual subject data, the stimulus-EMG cumulant density for the MVS-S exceeded the 95% confidence interval when using only two segments (i.e. 5 s) of data.

**Table 1:** Summary of signal-to-noise ratios (SNR) and cumulant density responses across input stimuli.

	Stimulus	SNR	Cumulant density responses			
			Latencies (ms)		Magnitudes	
			Short	Medium	Short	Medium
r-mGAS	SVS	0.11±0.08	61±4	101 ±5	0.060±0.012	−0.098±0.022
	MVS-S	0.40±0.01	62±3	97±3	0.058±0.011	−0.100±0.027
	MVS-4	0.46±0.07	63±3	98±6	0.039±0.012	−0.051±0.019
	MVS-L	0.50±0.10	58±3	101±6	0.037±0.012	−0.107±0.028
AP forces	SVS	0.09±0.01	130±13	261±34	−0.051±0.019	0.163±0.037
	MVS-S	0.32±0030.05	128±11	268±31	−0.050±0.021	0.184±0.046
	MVS-4	0.39±0.08	142±16	283±35	−0.047±0.014	0.078±0.038
	MVS-L	0.47±0.09	125±10	265±39	−0.055±0.030	0.259±0.044

Cumulant density responses include the short and medium EMG and AP-force latencies and magnitudes for each of the input stimuli (mean ± SD, n = 8).

**Figure 3:**
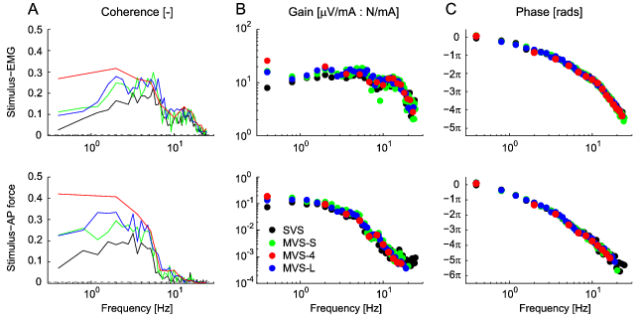
Coherence-, gain- and phase-frequency estimates of stimulus conditions for stimulus-EMG (top) and stimulus-AP force (bottom). A: pooled (n = 8) coherence plots for each stimulus condition. B: pooled (n = 8) gain plots for each stimulus condition. C: pooled (n = 8) phase plots for each stimulus condition. Gain and phase were plotted at frequencies with significant coherence and did not differ across stimulus conditions. Note the limited number of frequencies within the MVS-4 across the entire dynamic range and the limited number of frequencies in the MVS-L at high frequencies.

Significant stimulus-EMG coherence for the r-mGAS muscle was observed across the entire 0–25 Hz bandwidth for both SVS and all MVS signals (Fig 3A). For all signals, the stimulus-EMG coherence was characterized by two distinct peaks between 2–5 Hz and 11–17 Hz, and the stimulus-AP force coherence was characterized by a single peak between 2–5 Hz. In general, the coherence was higher for all MVS conditions at low frequencies (Fig. 3A); however, due to potential bias within MVS conditions no statistical comparison was made. Gain- and phase-frequency estimates for stimulus-EMG and stimulus-AP force demonstrated a low-pass behavior with cutoffs at ∼14 and 4 Hz respectively (Fig. 3B and C). Gain and phases were similar for all four signals indicating that variations in the stimulus frequency content do not modulate the frequency response of the underlying system.

**Figure 4:**
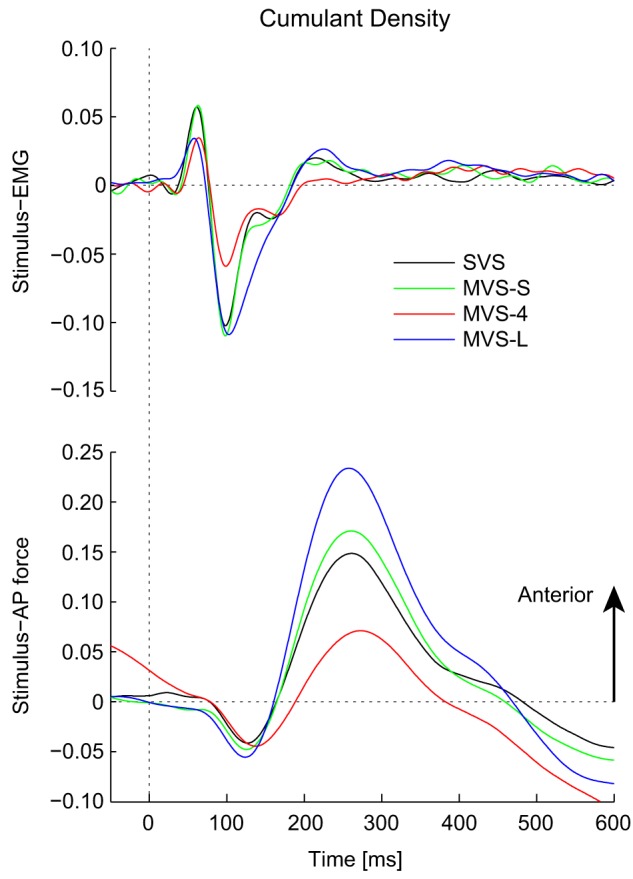
Pooled (n = 8) cumulant density estimates. Responses indicate a biphasic response for both the stimulus-EMG (top plot) and stimulus-AP force estimates (bottom plot).

Cumulant density estimates between stimulus-EMG and stimulus-AP force (Fig. 4) generated biphasic responses typical for stimuli that excite the full bandwidth of frequencies (0–25 ) [11], [12], [24]. For almost all timing and magnitude measures (short and medium latency) there were no significant differences between the MVS-S and SVS stimuli (see Table 1) for both the EMG (multiple 0.7<t_7_<1.9, 0.087<P<0.532) and AP force (multiple 0.2<t_7_<1.9, 0.097<P<0.818) cumulant density estimates. Only the medium latency AP force magnitude increased on average by 13% during MVS-S (t_7_ = 3.1, P = 0.017). With the exception of this measure, these results confirm the expectation that responses would not differ using frequency equivalent SVS and MVS stimuli (i.e. MVS-S). In contrast, MVS-L decreased the short latency stimulus-EMG peak magnitude by 28% (t_7_ = 30.2, P<0.001) but had no effect on the medium latency peak magnitude (t_7_ = 1.5, P = 0.191) compared to SVS. MVS-L also had no effect on the short latency stimulus-AP force peak magnitude (t_7_ = 0.3, P = 0.739) but increased the medium latency peak magnitude by 58% (t_7_ = 13.2, P<0.001) compared to SVS. These results partially confirm the expectation that the concentration of stimulus power at low frequencies during MVS-L would decrease short latency and increase medium latency response magnitudes compared to SVS.

**Figure 5:**
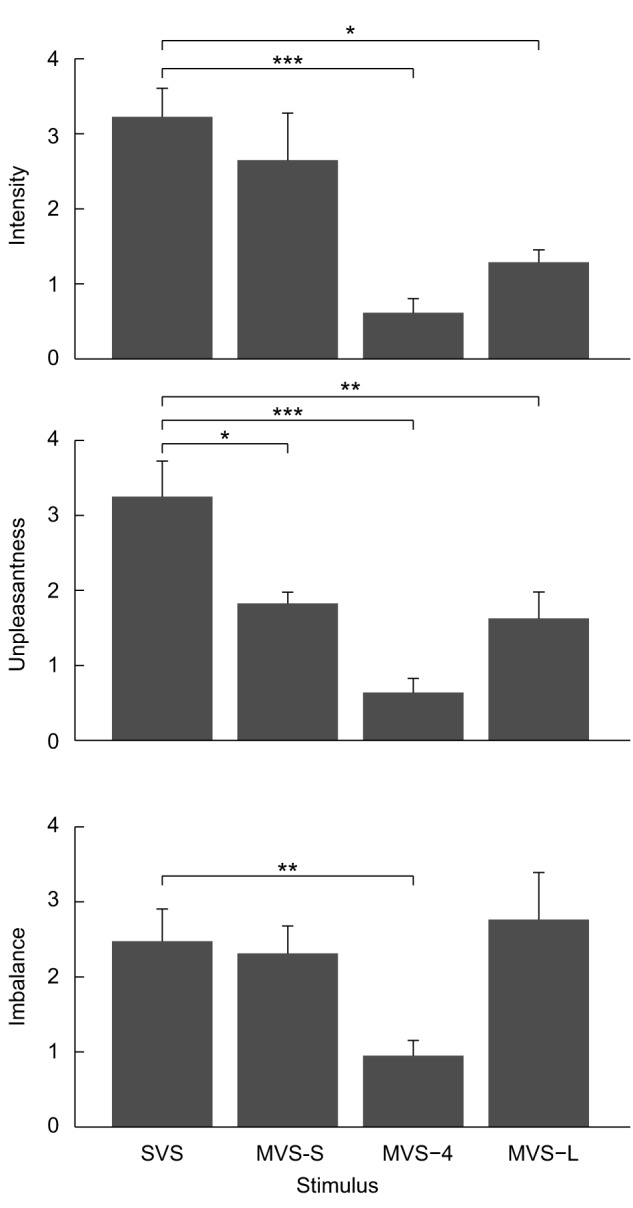
Reported psychophysical measures of intensity, unpleasantness and imbalance using visual analogue scales. Error bars are the standard error (n = 8). *  = P<0.05, **  = P<0.01, ***  = P<0.001 indicate significant differences as obtained from the pairwise comparison of SVS and all MVS stimulus conditions using a Bonferroni correction for multiple comparisons.

The subjective perception of intensity, unpleasantness and imbalance between the SVS and MVS signals differed (Fig. 5). Compared to SVS, the mean perceived intensity of the MVS-S, MVS-4 and MVS-L decreased by 18, 81 and 60% respectively (F_3,21_ = 11.0, P<0.001). Pairwise comparisons indicated that the decrease in intensity during MVS-4 and MVS-L was significant (MVS-4: P<0.001; MVS-L: P = 0.006) compared to the SVS stimuli but was not during MVS-S (P = 0.331). The absence of a significant difference for the MVS-S was attributed to one subject who rated the MVS-S intensity as 6.3 compared to all other subjects who rated it between 1.4–3.7. This was the only subject to rate MVS-S above SVS. The mean perceived unpleasantness for the MVS-S, MVS-4 and MVS-L stimuli decreased by 43, 80 and 50%, respectively, compared to SVS (F_3,21_ = 25.4, P<0.001) (Fig. 5). Finally, although the stimulus had a significant main effect on the perception of imbalance (F_3,21_ = 6.4, P = 0.003), the pairwise comparison revealed that only MVS-4 decreased subjects' perception by 62% compared to SVS (P = 0.004) but MVS-S and MVS-L were similar compared to SVS (multiple P = 1.000).

## Discussion

The primary aim of the study was to develop electrical vestibular stimuli that provide an accurate assessment of reflex responses while minimizing subject discomfort. The use of multisine vestibular stimulation (MVS) signals allowed us to distribute power over a precise number of frequencies within a desired bandwidth of interest (0–25 Hz). The limited number of frequencies (i.e., MVS-4 16 frequencies and MVS-L 35 frequencies vs. MVS-S 62 frequencies) and cresting process (i.e., all MVS vs. SVS) provided lower stimulus peak amplitudes thereby decreasing the subjective perception of stimulus intensity and unpleasantness. Furthermore, the SNR for MVS signals increased 3–6 times compared to SVS, thus requiring a reduced number of segments (i.e., time exposed to the stimulus) to provide reliable estimates of vestibulo-motor responses.

Reduced subject load (discomfort and time) and improved SNR can help reduce subject variability and is ideal when examining reflexes in research and clinical settings. For example, to investigate the modulation of vestibular reflexes during locomotion MVS-S would be advantageous over SVS, and according to our results would reduce the minimum number of steps required to evoke a reliable response from 250 to 38 [33], [34]. In a clinical setting, the reduced experimentation time provided by MVS-S would improve patient comfort since the majority of protocols use short duration square wave pulses [17], [20] that require numerous repeated pulses to estimate vestibular reflex and postural responses, and are often reported as painful. MVS signals should make electrical vestibular stimulation a more viable option for clinicians to assess patients with disturbed vestibulo-motor responses.

The frequency response (gain and phase) of both vestibular reflexes (EMG) and postural behaviour (AP force) did not vary across stimulus conditions, suggesting linear system behaviour regardless of the frequency content of the input vestibular stimulation. These findings extend those from Dakin et al. [27], who observed that stimulus-EMG cumulant density responses are a linear summation of all stimulated frequencies. More directly, our MVS-4 results confirm the suggestion of system linearity by demonstrating that nonlinear contributions were limited to <15% of the non-stimulated harmonic frequencies and were of a similar magnitude to the noise. Although not formally compared, the low frequency increase in coherence in all MVS stimulus conditions indicate that a larger component of the variability of the muscle activity and AP forces can be attributed to the variability in the applied stimuli (MVS) relative to SVS. As a result, the low coherences observed during the SVS signal are likely due to the high levels of noise present in the EMG signals and the small muscle responses elicited by electrical vestibular stimulation [8] rather than due to nonlinearities. Together, these results suggest that MVS signals that excite a limited number of frequencies, such as the MVS-4, can provide an appropriate linear estimate of the vestibulo-motor frequency responses.

The explicit selection of individual frequencies in the MVS signal allowed us to modulate either the short or medium latency component of the reflex and postural responses. The effects of removing high frequencies during MVS-L (i.e. decreased short latency stimulus-EMG magnitude and increased medium latency stimulus-AP force magnitude) did not exactly match the expected outcome (i.e. decreased short latency and increased medium latency of both stimulus-EMG and -AP force). However, the attribution of high frequencies to short latency responses and low frequencies to medium latency responses is not exclusive, since the resultant response is a linear composite of all stimulated frequencies [27]. Nevertheless, multisine signals such as the MVS-L demonstrate a key advantage over bandpass-filtered SVS approaches [11], [24] by allowing for complete and explicit control over the frequencies contributing to the evoked response. For example, evoking an increased medium latency component may be particularly useful when studying the net contribution of semicircular canals to electrically evoked vestibulo-motor responses [24]. Furthermore, a potential additional benefit of MVS signals not explored here is the ability to separately control the power of individual frequencies in the input stimulus [35]. By exploiting this potential advantage, the frequency response of the system could be characterized across the entire bandwidth with a high resolution while evoking targeted features of the vestibulo-motor response (i.e. short or medium latency) in the time domain.

There are limitations associated with MVS signals. First, the frequency responses can only be estimated at frequencies where the stimulus signal contains power. Therefore, the distribution of power over fewer frequencies reduces the resolution of the estimated system responses [23], and one has to balance between increased SNR and frequency resolution. Second, the signals are periodic and as a result the period of the analysis window is locked to the inverse of the lowest frequency or integer multiples of this period. Therefore, the number of segments used for averaging cannot be increased a posteriori in an effort to improve vestibulo-motor response estimates. Finally, although the MVS-4 was used to evaluate nonlinear distortions at non-stimulated frequencies, it did not allow for complete isolation of nonlinear distortions at all stimulated frequencies. This was due to inclusion of the first frequency point, which was a repeating integer of all other stimulated frequencies (i.e. harmonics). As a result, it is theoretically possible that the power at stimulated frequencies may have been a combination of linear contributions and nonlinear harmonic distortions. However, because the power at non-stimulated harmonic frequencies was not substantially larger than the noise, it is not expected that such confounding factors would influence our linear estimates of the frequency responses. Recent improvements in system identification methods Pintelon and Schoukens [36] can provide a more comprehensive assessment of system nonlinearities and are expected to produce similar outcomes to those found in this study.

In summary, we have demonstrated several advantages of MVS over SVS, including increased SNR, enhanced vestibulo-motor responses, reduced experimentation time and improved subject comfort. These improvements may be particularly beneficial for basic research applications or when studying subjects with disturbed vestibulo-motor processing. In addition, we have shown that MVS signals can be designed to assess system linearity and evoke targeted vestibulo-motor responses.

## Supporting Information

File S1 Experimental multisine vestibular stimulation signals (.txt files) and Matlab code (MVS_SignalPlotting.m) to plot the signals used in this study, as well as Matlab code to generate new signals (MVS_SignalGenerator.m).
(ZIP)
